# Translational control mechanisms in cutaneous malignant melanoma: the role of eIF2α

**DOI:** 10.1186/s12967-019-1772-z

**Published:** 2019-01-11

**Authors:** Immacolata Maida, Paola Zanna, Stefania Guida, Anna Ferretta, Tiziana Cocco, Luigi Leonardo Palese, Paola Londei, Dario Benelli, Amalia Azzariti, Stefania Tommasi, Michele Guida, Giovanni Pellacani, Gabriella Guida

**Affiliations:** 1 Department of Basic Medical Sciences Neurosciences and Sense Organs, University of Bari, Bari, Italy; 2 Dermatology Unit, Department of Surgical, Medical, Dental and Morphological Science with Interest Transplant, Oncological and Regenerative Medicine, University of Modena and Reggio Emilia, Modena, Italy; 3 Department of Cellular Biotechnology and Hematology BCE, Sapienza University of Rome, Rome, Italy; 4 National Cancer Research Centre “Giovanni Paolo II”, Bari, Italy

**Keywords:** Metastatic melanoma, eIF2α, BRAF, MiT family, Nuclear peIF2α, Structural analysis

## Abstract

**Background:**

Melanoma cells develop adaptive responses in order to cope with particular conditions of tumor microenvironment, characterized by stress conditions and deregulated proliferation. Recently, the interplay between the stress response and the gene expression programs leading to metastatic spread has been reported.

**Methods:**

We evaluated levels and localization of eIF2α/peIF2α in V600BRAF and wtBRAF metastatic melanoma cell lines by means of western blot and confocal microscopy analyses. Furthermore, we performed a sequence analyses and structure and dynamics studies of eIF2α protein to reveal the role of eIF2α and its correlations in different pathways involved in the invasive phase of melanoma.

**Results:**

We found peIF2α both in cytoplasm and nucleus. Nuclear localization was more represented in V600BRAF melanoma cell lines. Our studies on eIF2α protein sequence indicated the presence of a predicted bipartite NLS as well as a nuclear export signal NES and an S1 domain, typical of RNA interacting proteins. Furthermore, we found high levels of transcription factor EB (TFEB), a component of the MiT/TFE family, and low β-catenin levels in V600BRAF cells.

**Conclusions:**

Based on our results, we suggest that peIF2α nuclear localization can be crucial in ER stress response and in driving the metastatic spread of melanoma, through lysosomal signaling and Wnt/β-catenin pathway. In conclusion, this is the first evidence of nuclear localization of peIF2α, representing a possible target for future therapeutic approaches for metastatic melanoma.

**Electronic supplementary material:**

The online version of this article (10.1186/s12967-019-1772-z) contains supplementary material, which is available to authorized users.

## Background

Melanoma is one of the skin tumors with increasing incidence in the general population [1]. It is a particularly severe form of skin cancer due to its ability to metastasize, even several years after the removal of the primary melanoma, and to its resistance to medical therapies in case of metastasis. One of the peculiar characteristics of melanoma is the involvement of several molecular pathways in its development and progression. This feature is associated to diverse escape mechanisms to target therapies (i.e. anti-BRAF) for the metastatic disease.

The most recent knowledge of melanoma biology focuses on the role of endoplasmic reticulum (ER) stress, autophagy and translational reprogramming. In brief, tumor microenvironment conditions, characterized by stress *stimuli* and a push to fuel continue proliferation, induce the development of cellular adaptive responses. In particular, these stress signals induce the activation of the unfolded protein response (UPR) [2–4], and translational block, in order to avoid the accumulation of proteins entering the ER. The translational block is achieved through the phosphorylation of the alpha subunit of eukaryotic translation initiation factor 2 (eIF2α) which is one of the key regulator of the Integrated Stress Response (ISR). ISR is a common adaptive pathway, activated in response to diverse stress stimuli, in order to restore cellular homeostasis [5]. The central core of ISR response is eIF2, a heterotrimeric protein constituted by α, β and γ subunits, an essential factor involved in the initiation phase of eukaryotic translation. eIF2 is a G-protein that, in its active GTP-bound form, interacts with Met-tRNAi promoting its binding to the 40S ribosomal subunit and determining the correct localization on the start codon. The codon-anticodon interaction triggers GTP hydrolysis, whereby an inactive eIF2-GDP complex is released. eIF2 reactivation is catalyzed by the recycling factor eIF2B that allows GDP/GTP exchange. Indeed, the recycling of the factor can be inhibited by the phosphorylation of its alpha subunit at Ser 51. In its phosphorylated form, eIF2 behaves as a competitive inhibitor of eIF2B, preventing GDP/GTP exchange and thereby reactivation of eIF2. eIF2 phosphorylation is triggered by various stress conditions, such as viral infection or nutrient deprivation, which lead to the activation of specific kinases (PKR, PERK, GCN2, HRI). The phosphorylation of eIF2α determines an increase in the expression of the transcription factor ATF4, a key component of the ISR, in order to promote cellular recovery. ATF4 is a transcription factor of the ATF/CREB family that regulates the expression of genes involved in oxidative stress, amino acid synthesis, differentiation, metastasis and angiogenesis and it is induced by stress signals including anoxia/hypoxia, ER stress, amino acid deprivation, and oxidative stress [6].

There are evidences that eIF2α expression levels and phosphorylation status increase in tumours compared with normal tissue [7–11]. In melanoma, it has been previously demonstrated that in V600BRAF metastatic melanoma cell lines eIF2α is more phosphorylated, respect to wtBRAF melanoma cells [12].

A recent study [13] shows the important interplay between the ISR and the gene expression programs leading to metastatic spread, regulated by MITF [13]. MITF is a transcription factor that drives the expression of genes involved in cell differentiation, proliferation and survival. It plays an important role in melanocyte development by regulating the expression of genes implicated in melanin production and its levels have been associated to melanocytes transformation to melanoma [14, 15]. One of the components of the MITF/TFE family of transcription factor [16] is TFEB, well-known for its key role in autophagy activation and recently proposed as a master regulator of ISR [5].

Several pathways involved in cellular proliferation, translational reprogramming, together with molecular adhesion variations (i.e. β-catenin), seem to be the key events that drive gene expression to an invasive phase of melanoma [12, 13]. Because of its pivotal role in translation initiation, eIF2α is the main target of translational regulation mechanisms. Thus, the aim of the current work is to perform a detailed evaluation of eIF2α/peIF2α levels and location. In addition, we estimated the correlation of eIF2α with different pathways involved in the invasive phase of melanoma in an in vitro melanoma model.

## Materials and methods

### Cutaneous melanoma cell lines

Hmel1 and M3 were metastatic melanoma cell lines extracted in our laboratory and proceeding from patients coming from the south of Italy. These cell lines have been described in Zanna et al. [17], and Ferretta et al. [12]. Hmel1 and M3 represent the metastasis harboring V600BRAF mutation. HBL is a metastatic melanoma cell line, kindly provided by Ghanem Ghanem. It was used as control cell line because it is wt for BRAF. In addition, hmel9 cells, non-metastatic V600BRAF-mutated, previously described [12, 17] were used.

### Western blots and cell fractioning

Cells were lysed as described in Herraiz et al. [18]. Bicinchoninic acid reagent, from Sigma Aldrich (St. Louis, MO), was used to quantify proteins. Electrophoresis and Western blots were performed as described in  Ferretta et al. [12]. 30–50 µg of the cell lysate were loaded on a SDS-PAGE (10% or 13% for TFEB) and transferred to a nitrocellulose membrane (GE Healthcare, Little Chalfont, UK) for Western Blot analysis. The polyclonal antibodies, anti-eIF2α and anti-peIF2α Ser 51 were purchased from Cell Signaling Technology (Danvers, MA); the polyclonal anti βcatenin antibodies and the secondary antibody-HRP conjugated were purchased from Santa Cruz Biotechnology (Santa Cruz, CA). The mouse anti Flag M2 monoclonal antibody was from Sigma. TFEB antibodies and the anti-mouse HRP secondary antibody were from Millipore (Burlington, Massachusetts, US). Protein loading was assessed reprobing the blots with anti-βtubulin antibodies (Sigma Aldrich, St. Louis, US). All the antibodies were used according to the manufacturer’s instruction. Chemioluminescence was detected using the Super signal western Pico Pierce or the chemiluminescent Pierce ECL Western Blotting Substrate (Thermo Scientific, IL). The densitometric profiles were performed using a Image lab 5.2 imaging software (Bio-Rad Laboratories) or Bio-rad GS 800, and analyzed by the Quantity One Software, all purchased by Bio-Rad Laboratories (Hercules, CA).

The M3 cell fractioning was performed as previously described in Zanna et al. [19], obtaining nuclear and cytoplasmic fractions used for western blot analyses. The purity of the nuclear and cytosolic fractions was verified by the presence of βtubulin immunoreactive band after immunoblotting with anti-βtubulin antibody).

#### Confocal microscopy

Cells grown on coverslips and washed with PBS, fixed with 4% paraformaldehide, and blocked with 20 mM glycine. Cells were permeabilized with 0.5% Igepal CA-640 in PBS. Cells were stained with anti-eIF2a and the anti-peIF2α diluted in PBS containing 1% BSA. Goat anti-rabbit Alexa 488 conjugated antibody Molecular Probes (Invitrogen, CA) in PBS/1%BSA was used. Incubations were performed at 4 °C. Samples were examined with a Nikon confocal microscope (Nikon Corporation, Tokyo, Japan).

### eIF2α cloning in a pCMV-flag vector

eIF2α molecular cloning was performed on pCVM-flag (Sigma Aldrich, St. Louis, MO). eIF2α coding sequence was obtained by M3 cDNA by PCR using a proofreading polymerase (Kapa Hifi, Roche, Basilea Switzerland) and the primers eIF2αHINDIIFw (5′-CGCAAGCTTCCGGGTCTAAGTTGTAGA-3′) and eIF2αXbaIRv (5′-CGCTCTAGACACAAAGTTAATCTTCAGC-3′), purchased from Sigma. The PCR product and pCMVflag were digested for HindIII and XbaI restriction enzymes (Promega, Wisconsin, US), loaded on a 1% preparative agarose gel and eluted using the extraction kit purchased by RBC Biosciences (New Taipei, Taiwan). The ligation of the pCMV vector with the 947 bp PCR eIF2α was performed o.n. at 4 °C using a T4 DNA ligase (Promega) according to the manufacturer’s instructions. Ligations were used to transform Top10 chemically competent *E. coli* (Thermo Fisher Scientific, Waltham, Massachusetts, US), the colonies were selected and minipreps were screened by digestion with HindIII and XbaI. The pCMVflag and pCMVflag-eIF2α were used to transfect 293T cells using lipofectamine 2000 transfection reagent (Invitrogen, Meridian Rd, Rockford, IL), according to manufacturer’s instructions.

### eIF2α structural analysis

The solution NMR structure of the alpha subunit of human eIF2 1Q8 K [20] was obtained from the RCSB PDB [21] available at http://www.rcsb.org. From each of the reported models in the 1Q8 K entry a separate pdb file was obtained by Tcl scripting in VMD [22]. These pdb structures were processed as described in [23, 24]. Briefly, structures in the data set were aligned to a reference by a rotation and translation matrix, by a Tcl script in VMD. The Cartesian coordinates of alpha-carbon atoms of the rotated structures were extracted and arranged in a matrix form in which each row represents a structure, and principal component analysis (PCA) was performed on the normalized data [25–28]. To visualize the obtained principal displacement modes encoded in the whole 1Q8K PDB entry, a nmd file was constructed [23, 24], which was analyzed by the VMD plug-in NMWiz [29].

The prediction of nuclear export signals (NESs) in the sequence of the alpha subunit of human eIF2 has been performed at the NetNES 1.1 Server [30]. Protein–protein interactions were obtained from the BioGridrepository [31, 32].

The prediction of eIF2α nuclear localization signals (NLS) were performed using the program cNLS Mapper (available at http://nls-mapper.iab.keio.ac.jp/cgi-bin/NLS_Mapper_form.cgi) using a cutoff 4. An additional analysis of the eIF2α functional domains has been performed using the SMART program (http://smart.embl-heidelberg.de/).

## Results

### peIF2α localization in melanoma cell lines

**Fig. 1:**
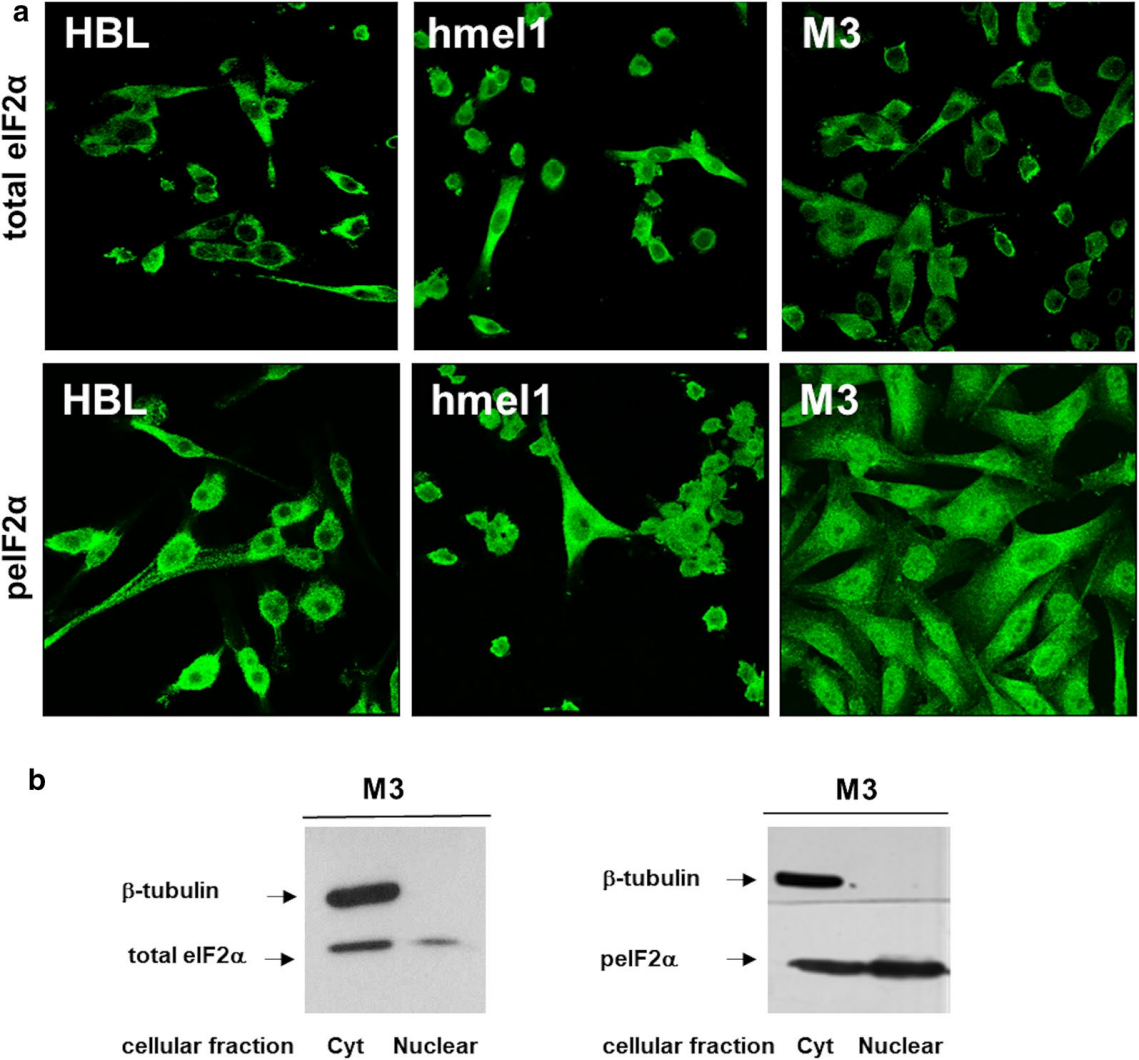
eIF2α and phosphorylated eIF2α (peIF2α) translation factor localization in HBL (wtBRAF), hmel1 and M3 (V600BRAF) metastatic melanoma cell lines. **a** Confocal microscopy of localization of eIF2α and peIF2α using eIF2α (total form) or peIF2α (S51) antibodies in HBL (wtBRAF), hmel1 and M3 (V600BRAF) metastatic melanoma cell lines. **b** Western blotting analyses using eIF2α (total form) or peIF2α (S51) antibodies were performed on M3 nuclear and cytoplasmic fractions. The spot of β-tubulin demonstrates the purity of the nuclear and cytoplasmic fractions

In the present work, in order to provide meaningful information about the subcellular localization of peIF2α and eIF2α, we performed studies with specific antibodies against the phosphorylated (peIF2α) and the total eIF2α form (eIF2α) comparing metastatic melanoma hmel1 and M3 (V600BRAF) with HBL cell lines (wtBRAF). The analysis of the eIF2α subcellular localization revealed a prevalent cytoplasmic localization, as compared to the nuclear compartment without significant differences among the different metastatic melanoma cell lines (Fig. 1a). Analysis of the peIF2α, beyond the cytoplasmic localization, revealed a nuclear staining in all the melanoma cell lines (Fig. 1a). This nuclear localization was more evident in V600BRAF M3 and hmel1 melanoma cell lines (Fig. 1a), as compared to wtBRAF HBL. To avoid the possibility that such result could be due to a non-specific antibody background, we performed a M3 cell fractioning separating the cytoplasmic from the nuclear fraction. The western blotting analysis using total eIF2α or peIF2α antibody on the cytoplasmic and nuclear M3 cellular fractions confirmed that the peIF2α was present both in the nucleus and in the cytoplasm (Fig. 1b), even if the total eIF2α was more abundant in the cytoplasmic fraction. The purity of the fractions was checked by SDS-PAGE and western blotting analysis using an anti β-tubulin antibody (Fig. 1b).

As reported in Ferretta et al. [12] the non-metastatic V600BRAF hmel9 cell line, showed a lower level of peIF2α, as compared to metastatic melanoma cells [12]. Therefore, we extended the analysis of peIF2α cytoplasmic and nuclear localization to hmel9 cell line, finding lower nuclear level of peIF2α in non-metastatic V600BRAF hmel9 cells, as compared to metastatic V600BRAF M3 cells (see Additional file 1: Fig. S1).

### eIF2α sequence analysis and eIF2α-flag expression and localization in 293T transfected cells

**Fig. 2:**
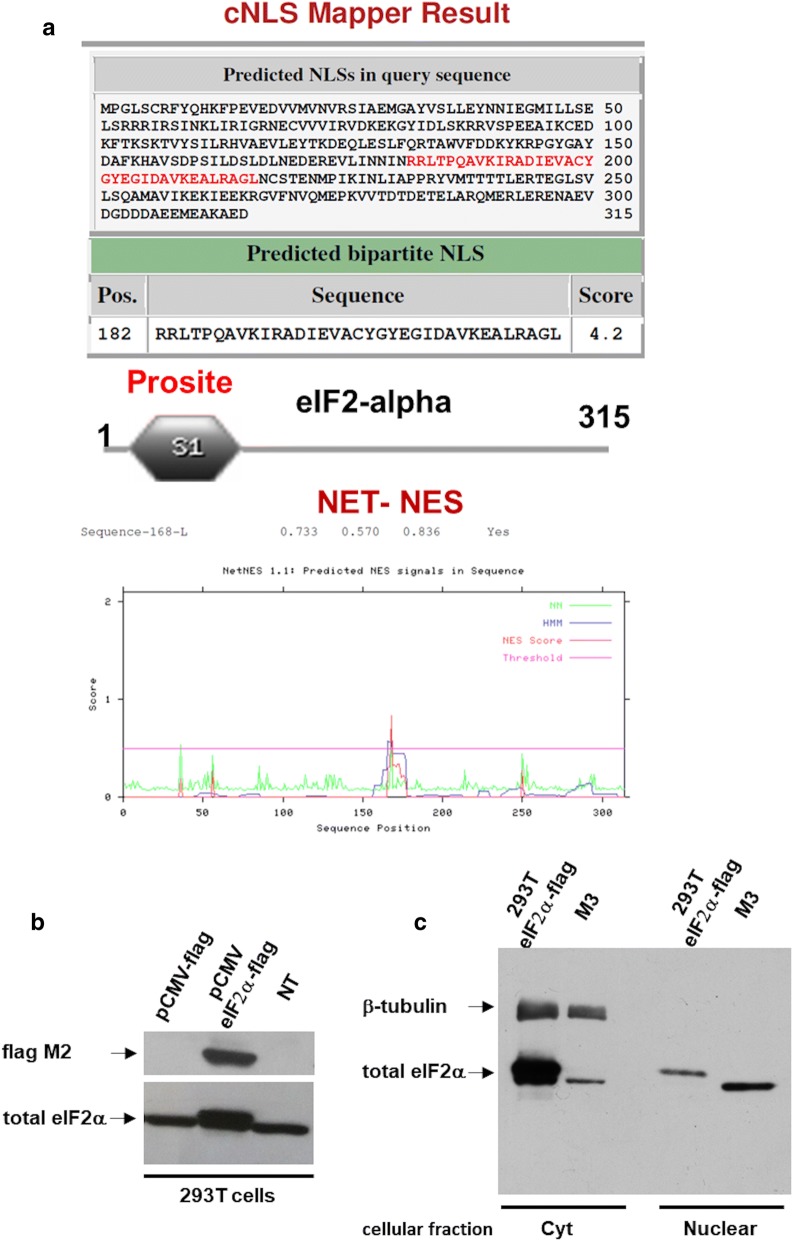
eIF2α sequence analysis and eIF2α-flag expression and localization in 293T transfected cells. **a** eIF2α sequence analysis using the program cNLS mapper. The sequence analysis indicated the presence of a predicted bipartited NLS from the amino acid position 182 to the 216. SMART program identify an S1 domain, typical of proteins interacting with RNA. NES analysis using the NetNES 1.1 Server revealed the presence of one nuclear export signal localized at position 168. **b** Western blotting of the total cell extract from 293T cells transfected with a pCMV-flag vector, pCMV-flag-eIF2α vector and not transfected cells stained with anti-Flag M2 antibody and anti-total eIF2α antibody. **c** Western blotting of the cytoplasmic and nuclear fraction from 293T cells transfected with pCMV-flag-eIF2α vector and M3 melanoma cells, stained using an anti-total eIF2α antibody. β-tubulin antibody was used to demonstrate the purity of the nuclear and cytoplasmic fractions

We investigated the eIF2α sequence to detect if there were some signal sequences for its entrance into the nucleus using the program cNLS mapper (http://nls-mapper.iab.keio.ac.jp/cgi-bin/NLS_Mapper_form.cgi). The sequence analysis indicated the presence of a predicted bipartited NLS from the amino acid position 182 to the 216 with a score of 4, 2 (Fig. 2a) which suggests the presence of a nuclear transporter that is able to recognize and bind eIF2α. Moreover, NES analysis using the NetNES 1.1 Server (http://www.cbs.dtu.dk/services/NetNES/) revealed the presence of one nuclear export signal localized at pos 168 (Fig. 2a). Using SMART program, we found an S1 domain typical of proteins interacting with RNA [33].

A further evidence of eIF2α nuclear localization was achieved generating a construct in which eIF2α was expressed in the pCMV-flag vector under the control of the cytomegalovirus promoter and transfected in 293T cells. We analyzed the total extracts of the 293T transfected with the pCMVflag-eIF2α expression vector in comparison with 293T cells transfected with the empty pCMV-flag vector. The exogenous eIF2α protein was expressed and recognized by western blotting using anti flag M2 antibody (Fig. 2b). To detect if eIF2α was transported from the cytoplasm (site of translation) into the nucleus, we isolated transfected 293T cytoplasmic and nuclear fractions (Fig. 2c). We performed a western blot using the anti-eIF2α antibody and we detected eIF2α-flag either in the nucleus and in the cytoplasmic fraction of the 293T transfected cells. The eIF2α-flag is expressed and recognized by the anti-eIF2α antibody (total form) like a slightly higher molecular weight protein, due to the presence of the flag, with respect to the endogenous eIF2α as shown in M3 cell line used as control (Fig. 2c). The fractions purity was determined using βtubulin as cytoplasmic marker (Fig. 2c).

### eIF2α structure and dynamics

**Fig. 3:**
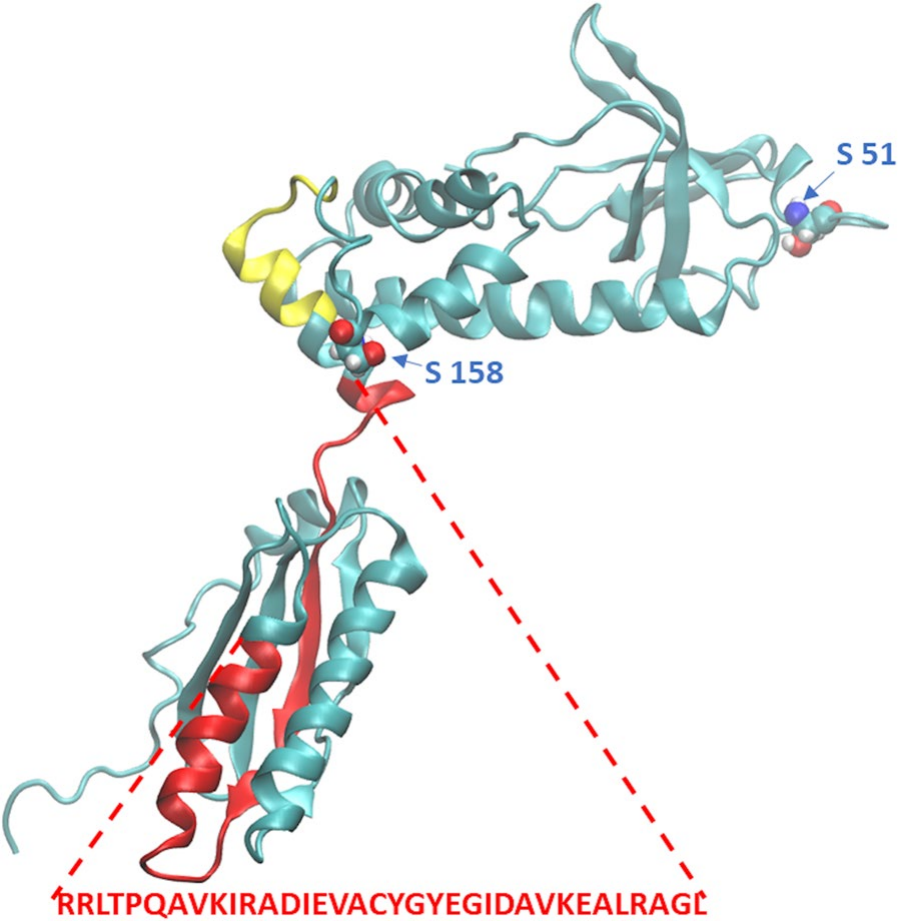
Structure of the α subunit of the eIF2α. The figure shows only one model of the NMR solution structures reported in the PDB entry 1Q8 K. A predicted NLS is reported in red, while the NES is reported in yellow. The serine residues (S51, S158) discussed in the text are highlighted as van der Waals surfaces

The solution structure of human eIF2α shows that the protein consists of two domains [20]. The N-terminal domain contains two distinct subdomains, an S1-type oligonucleotide/oligosaccharide binding fold and an alpha-helical region. The C-terminal domain has an alpha–beta fold that is similar to the C-terminal domain of the translation initiation factor eIF1B. These two domains are mobile relative to each other. Principal mode analysis of the deposited structure shows that the domains can rotate relative to one another exhibiting a twisting motion (see Additional file 2: Model 1). Moreover, a bending movement can be observed in which the two domains tend to approach (or move away) from one another (this is reported as Additional file 3: Model 2). Interestingly, recent data suggest that this last movement can be such as to bring the two domains into contact, and this closed conformation is disrupted by phosphorylation of S51 [34]. A predicted bipartite nuclear localization sequence (NLS) could be partially masked in the closed conformation of the protein. This region is reported in red in Fig. 3. The protein contains also a region where a (weak) nuclear export signal (NES) is expected (in yellow in Fig. 3). However, the functional significance of this NES is corroborated by the observation that a physical interaction between the protein and XPO1 has been reported experimentally [35]. Furthermore, a phosphorylation position is near this region (S158; the residues of serine that can be phosphorylated are highlighted in Fig. 3).

### TFEB and βcatenin expression analysis

**Fig. 4:**
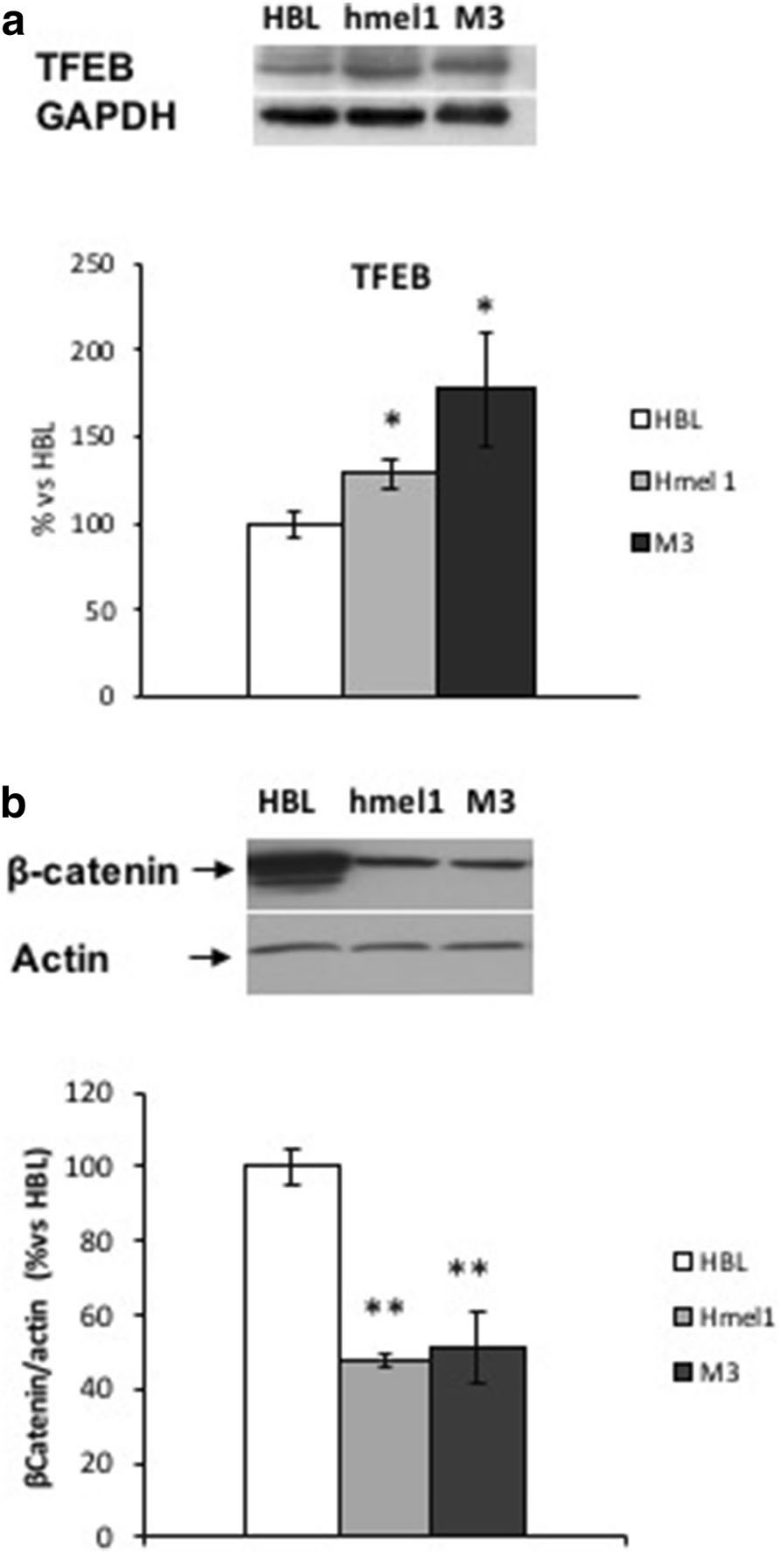
Western blotting analyses of transcription factor TFEB and βcatenin, performed on whole lysates from HBL (wtBRAF) and hmel1 and M3 (V600BRAF) metastatic melanoma cell lines. **a** Bar graph shows quantification of TFEB western blotting densitometric profiles normalized for GAPDH as loading control and HBL as control cell line. **b** Bar graph shows quantification of β-catenin by densitometric profiles normalized for β-actin used as loading control and HBL as control cell line. Values are mean ± SEM of three independent experiments. Significance was calculated with Student’s *t* test; *p < 0.05, **p < 0.005 vs HBL

Since MITF/TFE family factors can regulate lysosomal signaling, including Wnt/β-catenin [36] we studied TFEB and βcatenin levels in V600BRAF melanoma cell lines. The transcription factor TFEB has been shown to control lysosomal biogenesis and autophagy by regulating the expression of several lysosomal and autophagy-related genes [37]. We analyzed basal levels of TFEB by semi-quantitative western blotting analyses in HBL, hmel1 and M3 melanoma cell lines (Fig. 4a). We observed higher TFEB levels in hmel1 and M3 V600BRAF melanoma cell lines, as compared to HBL wtBRAF cells, in accordance with our previous data [12] reporting higher levels of autophagy in V600BRAF melanoma cell lines with respect to wtBRAF cells.

We have already reported lower levels of βcatenin in various V600BRAF melanoma cell lines as compared to HBL wtBRAF cells [19]. In order to estimate β-catenin levels also in M3 cell lines, western blotting analyses were performed. In Fig. 4b we show a 2-times lower levels of βcatenin in V600BRAF hmel1 and M3 cell lines as compared to HBL cells.

## Discussion

In this study, we analyzed phosphorylation, localization and conformational variations of the eIF2α subunit in metastatic melanoma cell lines with different BRAF mutational status, evaluating potential correlation with other pathways involved in the invasive phase of melanoma.

We have previously shown that peIF2α level is higher in cell lines proceeding from skin metastases (hmel1 and M3) than in cell lines from non-metastatic skin lesions (hmel9 and hmel11), harboring the V600BRAF mutation [12]. As a matter of fact, increased levels of peIF2α have been related to a worst melanoma prognosis [12] and to the invasive phase of melanoma [13].

The most striking result of the current work is the finding, in metastatic cell lines, of a nuclear localization of peIF2α, as compared to non-metastatic cells (Additional file 1: Fig. S1).

Since eIF2α is well characterized as a translation initiation factor, one would expect to find it in the cytoplasm and not in the nucleus. Nevertheless, another translation initiation factor, eIF4E, has already been reported to have a nuclear localization [8, 38–40]. During conditions of low nutrient availability, eIF4E can be sequestered by eIF4E-binding proteins (4E-BP), thus limiting the assembly of the eIF4F complex [41]. The nuclear import of eIF4E is mediated by the eIF4E-transporter, binding both eIF4E and the nuclear import receptors, importin α/β [38, 42]. In the nucleus, eIF4E is known to promote the nuclear export of a set of mRNAs [42–45].

In this study, the nuclear localization of eIF2α is corroborated by the finding that the protein sequence contains signals for both import in, and export from, the nucleus. Furthermore, structural studies indicate that these signals are exposed and available in the phosphorylated form of the protein (Fig. 3 and Additional file 2: Model 1, Additional file 3: Model 2), justifying the observation that the nucleus is especially enriched in peIF2α. Indeed, recent data suggest that the two domains of the eIF2α may move toward or away from each other, switching from a closed to an open conformation. However, the closed conformation is disrupted by phosphorylation of S51 [34], keeping the protein in a conformational “opened” status that exposes the nuclear signals necessary for the entrance and the exit from the nucleus. In addition, our results suggest that the transport of this factor out of the nucleus could be due to the interaction with NES sequence of the same factor with XPO1, as recently reported experimentally by Kirly [35], a well-known nuclear exporter of cellular proteins and RNAs.

The significance of peIF2α in the nucleus is still unclear. However, it is known that eIF2α can be phosphorylated by several kinases, activated in order to counteract adverse cellular conditions in tumors. Accordingly, tumor cells develop adaptive responses in order to cope with particular conditions of the tumor microenvironment, characterized by stress *stimuli* and a push to fuel continue proliferation. These stress signals induce the activation of the unfolded protein response (UPR) [2–4], which enables the accumulation of misfolded or unfolded proteins in the ER. Responses include an enhanced protein refolding, degradation of misfolded proteins, and translational block, in order to avoid the accumulation of proteins entering the ER. Therefore, ER stress, leading to eIF2α phosphorylation, inhibits eIF2B, which blocks the assembly of the ribosome translation initiation complex, thus reducing the rate of translation initiation [13, 46]. We have already demonstrated that eIF2α phosphorylation and ERK phosphorylation, due to constitutive activation of BRAF signaling in V600BRAF-mutated cell lines, are correlated. Furthermore, we also found different MITF levels in melanoma cell lines harboring BRAF mutations, as compared to wtBRAF. Accordingly, lower MITF levels in V600BRAF cells [12] (Fig. 3a) can be explained by the recently described transcriptional/translational coupled feedback loop in which MITF increases ATF4 transcription, and peIF2α promotes its translation. After that, MITF transcription and translation are blocked, respectively, by ATF4 and peIF2α-mediated inhibition of eIF2B [13].

On the other hand, ATF4 upregulation is also induced by TFEB, a component of the MITF/TFE family of transcription factors [16]. TFEB has a pivotal role in ER stress response and autophagy induction [47]. The high TFEB expression in V600BRAF metastatic melanoma cell lines found in the present work can be correlated with the high LC3II/LC3I ratio reported in Ferretta et al. [12], in the same cell lines, supporting the role of TFEB in autophagy.

Since MITF/TFE factors can regulate lysosomal signaling, including Wnt/β-catenin [36], we analyzed β-catenin levels. We found low β-catenin levels in V600BRAF cell lines, which can be correlated to the low MITF levels found in the same cell lines, as reported in Ferretta et al. [12]. Literature reveals that signaling through β-catenin enhances MITF expression. On the other hand, MITF, depending on its protein levels, can interact with β-catenin (alone or in a complex with Lef-1) to activate downstream targets [48]. The presence of low levels of β-Catenin, associated with low MITF levels in V600BRAF melanoma cell lines, could be linked to autophagy activation in these cell lines [49], as proved by the high TFEB levels revealed in this study. Interestingly, I.J. Davis group reported that TFEB can rescue MITF knockdown in cell sarcomas [50]. According with this study, our results indicate that MiT family members can compensate each other for oncogenic response in V600BRAF cells.

Translational reprogramming has been shown to drive gene expression programs leading to metastatic spread [13]. Therefore, eIF2α could affect the transcription of specific genes, and/or mediate the transport of specific mRNAs to the cytoplasm. In addition, eIF2α possesses a putative RNA-binding domain, and it has been suggested to interact with RNA polymerase [51]. Further studies will be performed to evaluate the nuclear eIF2α activity and to improve the emerging knowledge relating translational reprogramming to the invasive phase of melanoma, in order to contribute to the development of new target treatments for metastatic melanoma.

## Conclusions

This is the first report of the nuclear localization of peIF2α in metastatic melanoma cell lines.

Our structural studies of eIF2α protein sequence show the presence of a predicted bipartited NLS as well as NES and an S1 domain. These findings suggest its entry and exit in/from the nucleus and its role as a modulating factor of oncogenic pathways, especially implicated in establishing and maintaining a metastatic phenotype.

Taken together, our results underpin the key role of peIF2α nuclear localization in ER stress response and in the invasive phase of melanoma.

## Additional files

**Additional file 1: Fig. S1.** Phosphorylated eIF2α (peIF2α) translation factor in V600BRAF cells respectively metastatic (M3) and non-metastatic (hmel9) melanoma cell lines.A: Confocal microscopy of localization of peIF2α using peIF2α (S51) antibodies.B: Western blotting analyses using peIF2α (S51) antibodies were performed on M3 and hmel9 nuclear and cytoplasmic fractions. The spot of β-tubulin demonstrates the purity of the nuclear and cytoplasmic fractions.

**Additional file 2: Model 1.** Principal mode analysis of the deposited structure shows that the two domains of eIF2α can rotate relative to one other, exhibiting a twisting motion.

**Additional file 3: Model 2.** A bending movement can be observed in which the two domains of eIF2α tend to approach (or move away) from one other.
