# Supplementary figures and images for: Translational control mechanisms in cutaneous malignant melanoma: the role of eIF2α
Source: J Transl Med. 2019 Jan 11;17:20. doi: 10.1186/s12967-019-1772-z (PMC6329103; doi:10.1186/s12967-019-1772-z)

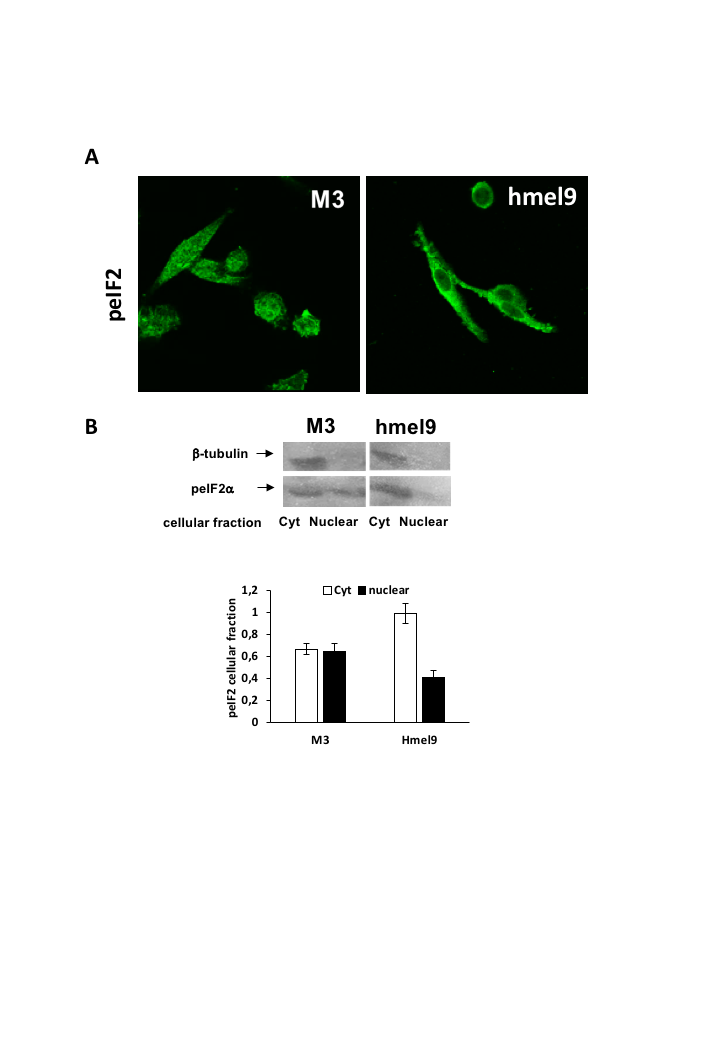

Supplement: Supplementary file 1 — Additional file 1: Fig. S1. Phosphorylated eIF2α (peIF2α) translation factor in V600BRAF cells respectively metastatic (M3) and non-metastatic (hmel9) melanoma cell lines.A: Confocal microscopy of localization of peIF2α using peIF2α (S51) antibodies.B: Western blotting analyses using peIF2α (S51) antibodies were performed on M3 and hmel9 nuclear and cytoplasmic fractions. The spot of β-tubulin demonstrates the purity of the nuclear and cytoplasmic fractions. [file 12967_2019_1772_MOESM1_ESM.tiff]

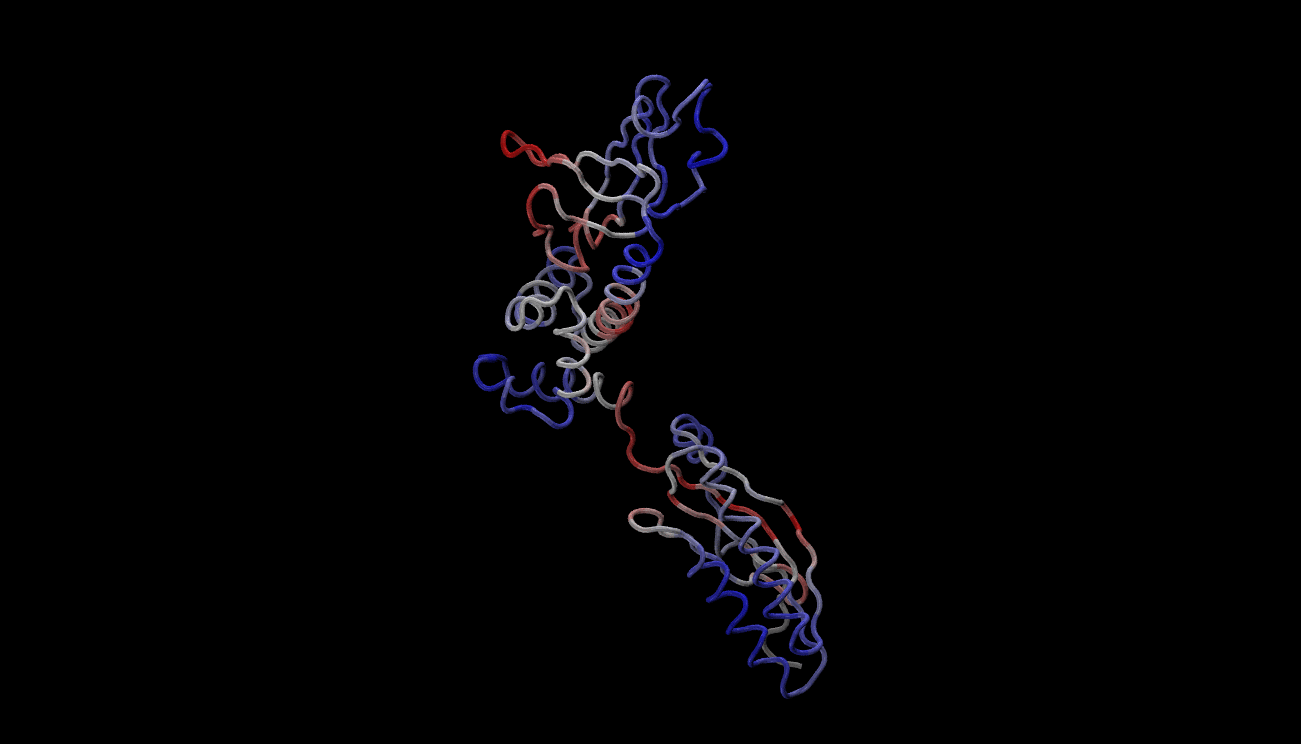

Supplement: Supplementary file 2 — Additional file 2: Model 1. Principal mode analysis of the deposited structure shows that the two domains of eIF2α can rotate relative to one other, exhibiting a twisting motion. [file 12967_2019_1772_MOESM2_ESM.gif]

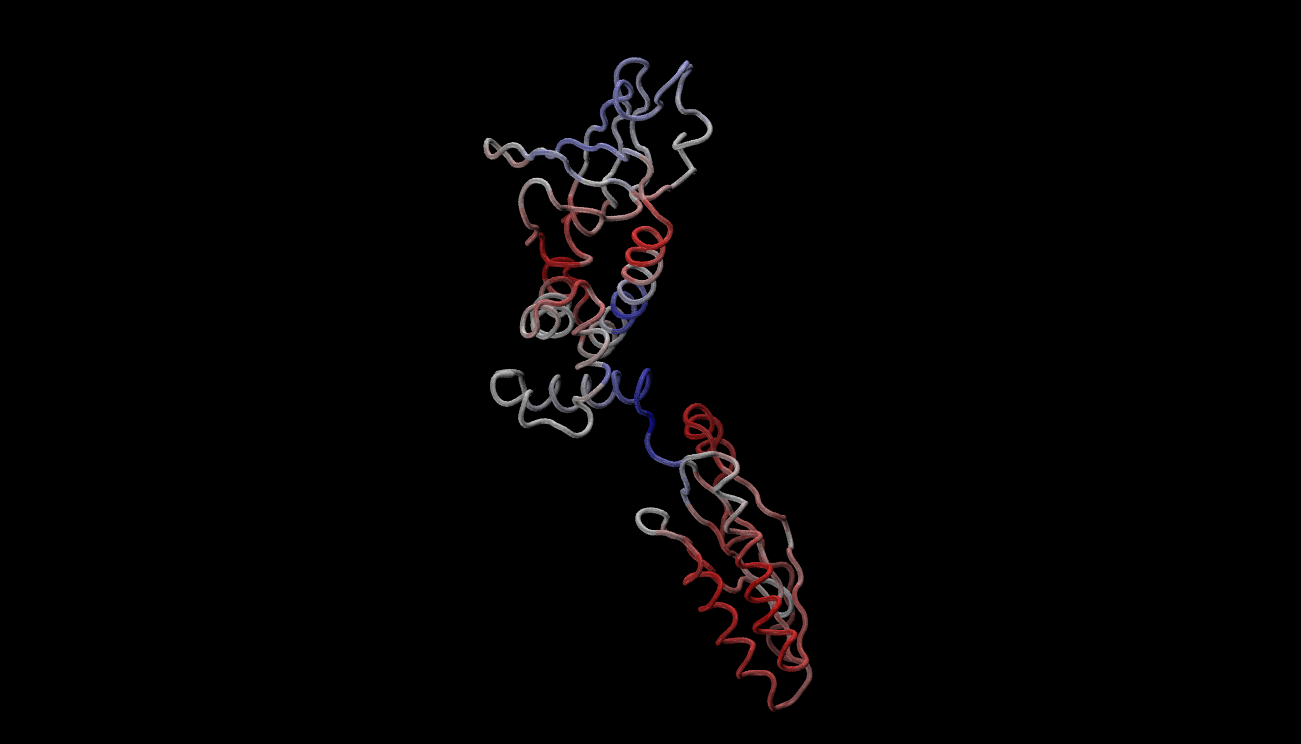

Supplement: Supplementary file 3 — Additional file 3: Model 2. A bending movement can be observed in which the two domains of eIF2α tend to approach (or move away) from one other. [file 12967_2019_1772_MOESM3_ESM.gif]
